# Climate-smart agriculture for sustainable agricultural sectors: The case of Mooifontein

**DOI:** 10.4102/jamba.v10i1.492

**Published:** 2018-01-31

**Authors:** Jennifer A. Mathews, Leandri Kruger, Gideon J. Wentink

**Affiliations:** 1 African Centre for Disaster Studies, North-West University, South Africa

## Abstract

Climate change is an environmental phenomenon with the potential to exacerbate existing disaster risks and cause extensive human, financial and environmental losses. The Mooifontein agricultural region in South Africa is considered to be a region vulnerable to climate change–associated risks. These climate risks would pose a substantial threat to the livelihoods of farmers in the Mooifontein area. This article aims to explore climate-smart agriculture (CSA) as a resilience-building tool to ensure sustainable agricultural practices. A qualitative research approach was utilised to gain insights into climate change and the lived experiences of farmers and agricultural experts in Mooifontein. The findings revealed that agricultural communities should focus on the identification and application of adaptation strategies like CSA. The adoption of appropriate CSA practices will play a vital role in ensuring sustainable livelihoods and improved community resilience for farming communities in the Mooifontein region.

## Introduction

Agricultural communities depend on their inherent knowledge of the seasons for decision-making about land use, crop selection, planting dates and seasonal rainfall patterns (Iloka 2016). Climate change risk holds threatening implications for the environment, ecosphere and natural order, resulting in negative impacts for farmers and their farming activities (Mathews 2016). Climate change risk threatens to disrupt the traditional practices and livelihoods of all humankind, but in particular those of rural communities and those who earn their living from the land (IPCC 2014:16, 65). Particularly for crop production, climate change risk poses a threat by accelerating soil erosion and degrading soils as a result of intense and frequent flooding due to climate change. This will cause lower yields and compromise food security (Lal et al. 2011:276). Other threats that climate change risk poses include drought and increased exposure to diseases, pests and weeds. This article defines climate change as ‘a change in climate that persists for an extended period and is caused either by natural processes or human activity’ (IPCC 2007:6). The impact that climate change has on agricultural production requires farmers to adopt adaptation strategies in order to reduce losses. Adaptation in the climate change context has been defined by Kropp and Scholze (2009:8) as adjustments that are made to natural and human systems as a response to actual and expected changes in climatic conditions in an attempt to limit damage or exploit opportunities. Similarly, disaster risk reduction (DRR) is considered to be a methodical process that pursues the design, and implementation of an integrated combination of appropriate policies, strategies and practices which is aimed at pre-empting hazardous situations and strategically lowering vulnerabilities in social systems to attain sustainable development (Mercer 2010:2). Both DRR and Climate Change Adaptation (CCA) target the reduction of risk and the impact of shock and aim to build resilience. The Hyogo Framework for Action (HFA) 2005–2015, which is considered as the primary international policy of DRR, was explicit about the need to integrate climate change mitigation and adaptation into DRR processes (Mitchell, Van Aalst & Villanueva 2010:11).

DRR practitioners acknowledge the importance of understanding the risk posed by climate change within a socio-economic framework, in particular where human activity might be exacerbating levels of vulnerability. It is essential to employ adaptation processes to increase the resilience of the lives and livelihoods of those most vulnerable to risks associated with climate change. These should be addressed through region-appropriate adaptation as part of national development agendas. The aim of this article is to explore adaptation opportunities using appropriate climate-smart farming systems that could build the resilience of the Mooifontein agricultural community in the North West Province (NWP) of South Africa.

### Demarcation of the study area

This article focuses on the Mooifontein agricultural region of the NWP of South Africa. Mooifontein covers approximately 10 000 ha including the villages of Brooksby, Mooifontein, Lombaardslaagte, Deelpan, Enselrus, Weltevrede and Uitkyk. Agriculture is integral to the economic development of the NWP and plays an important role in poverty alleviation. According to the NWPG (2008:237), 8.8 million hectares of the province consist of agricultural land of which 34.9% is arable and 56% is veld. The region is semi-arid and experiences mainly summer rainfall with an average of 360 mm per annum. The rainfall is erratic and is accompanied by high summer temperatures and high evaporation. Soils are sandy and susceptible to water and wind erosion (Bachtiar et al. 2003:xv). Agricultural activities in the Mooifontein region are primarily focused on animal husbandry, raising poultry and the cultivation of maize and sunflower crops. Most animals run on communal grazing lands that are not well managed (NWPG 2013:129). The Mooifontein region does not have adequate water supply for the purpose of agricultural activities (Getchell et al. 2002:179). Water for the purpose of crop irrigation and other agricultural activities is primarily sourced from boreholes, some of which have had to be drilled as deep as 100 m (NMMDM 2015:42).

Research revealed that a large percentage of the farmers in the Mooifontein region still practise monoculture, a system in which the same crop is planted every year, rather than following a crop rotation plan (Botlhoko & Oladele 2013:201). This increases disease and pest damage (Ncube et al. 2012:1) and probably contributes to the low productivity in the region (Botlhoko & Oladele 2013:202). Common farming enterprises range from vegetables, poultry, piggeries and livestock to cropping (Botlhoko & Oladele 2013:203). Farmers’ level of education is significant for adaptation education because it has been suggested that literate individuals are more likely to accept new innovations and contribute to more sustainable farming enterprises than illiterate farmers (Botlhoko & Oladele 2013:202; WRC 2013:25).

Climate change risk holds threatening implications for the environment, ecosphere and biodiversity and will impact farmers and farming activities in the Mooifontein region negatively. Patterns of land use and land cover will change over time because of climatic changes, which will threaten rangelands and grazing for livestock (NWPG 2014:23, 24). Areas where maize was traditionally planted will shift and maize-growing potential will decline (NWPG 2014:24). Sustainable livelihoods will be directly affected, which will in turn affect the general socio-economic status of diverse communities in the Mooifontein region. These insights demand that priority be given to adaptive management practices and skills and fresh approaches to addressing the uncertainty accompanying a changing climate and climate risk management (Vogel, Colvin & Scharfetter 2013:4). One such adaptive approach is embodied by climate-smart agriculture (CSA).

### Impacts of climate change in the agricultural sector

Globally, the impacts of climate change hold risks for agricultural practices, especially those countries where communities’ livelihoods are dependable from the land. The United Nations Framework Convention on Climate Change (UNFCCC) emphasised that climate change is linked to development and special attention should be given to developing countries that are most vulnerable to disasters (UNFCCC 2007). The African continent is most vulnerable to climate change and variability, increasing the hardships experienced by vulnerable communities (O’Brien et al. 2008:10; Scholtz 2011:11). By 2020, climate change will have impacted the lives and livelihoods of approximately 250 million people in Africa (Scholtz 2011:11). It will result in increased pressure on water resources, lower crop production and compromise in livelihoods and food security. In semi-arid countries like South Africa, moderate increases in annual temperatures will negatively impact water resources and impact crop production. South Africa is a semi-arid country with 14% of its surface area potentially arable (DEA 2011:91). Primary agriculture contributes 3.7% to the country’s gross domestic product (GDP), whilst agricultural workers make up 7.5% of the country’s formal employment. Economically, this sector comprises a highly developed commercial sub-sector geared towards export and a smaller subsistence sector – though there is much diversity and fluidity within these categorisations (Oladele 2011:92). Any change in the ‘climatic drivers’ of this sector could significantly impact production, employment opportunities and foreign earning potential (DEA 2015:67; Scholtz 2011:17, 18).

Water is considered ‘the primary medium through which climate change impacts will be felt by people, ecosystems and economies’ (Stuart-Hill, Schulze & Colvin 2012:1). Natural vegetation will change as climatic zones shift (Scholtz 2011:17–18) and the effect will be a shift in patterns of land use and changes in crop type, affecting the economy and communities’ livelihoods. South Africa’s temperature has increased over the past four decades, with marked increases in mean annual temperatures (Ziervogel et al. 2014:605). The annual rainfall trends reflect a general decrease in the number of rain days, an increase in intensity of rainfall and more prolonged dry spells (DEA 2013:3). The average rainfall of South Africa is 450 mm/year, which is significantly lower than the global average of 860 mm/year (Benhin 2008:666). South Africa’s Department of Water Affairs has warned that the impact of climate change on water supply varies, with changes in rainfall predicted with respect to their magnitude and variability.

Predictions for the coming years regarding South Africa’s climate are that climate change will result in lower rainfall and higher temperatures with increased flooding and drought events (DEA 2012:224–225). Changes in the distribution and availability of water will change agricultural patterns and more floods and droughts are likely to occur (DEA 2006:5). Climate change poses a serious threat and much of South Africa’s water sources and food production systems are facing challenges because ecosystems and biodiversity are also changing (Ziervogel et al. 2014:606).

The changes that have manifested highlight vulnerabilities and if left unmanaged will result in heightened vulnerability because the likelihood exists that recovery times between disaster events will be shorter, and the impact of these events on communities will be greater (Wisner, Gaillard & Kelman 2012:210). A ‘business as usual’ approach will neither stabilise food security nor secure a sustainable environment (Stern 2006:35, 143). For this reason, the HFA 2005–2015 explicitly stated that climate change mitigation and adaptation should be integrated into DRR processes (ISDR 2005:15; Mitchell et al. 2010:11). CSA is one of the DRR approaches identified to address the impacts experienced by climate change.

### Climate-smart agriculture

New management approaches are essential for the sustainable utilisation of natural resources. CSA has been explored as a DRR tool because it became apparent that more sustainable farming systems need to be identified to ensure sustainable livelihoods and improve disaster resilience (FAO 2010). In 2010 CSA was first defined by the FAO at the Global Conference on Agriculture, Food Security and Climate Change as an agricultural system, which increases productivity and sustainability. It enhances resilience by adapting to the effects of climate change, and can be considered as an approach that facilitates improved food security, creating an enabling environment in which sustainable development goals can be met (FAO 2010:ii). CSA should not be considered as a one-size-fits-all approach, but rather as an action plan uniquely designed to a location (Lima 2014:10) encompassing technology, policy and financial processes into development planning. CSA is therefore an integrated approach requiring the identification and implementation of sustainable agricultural development processes as a direct response towards climate change (FAO 2013:ix). It aims to sustainably address climate change issues through the guidance of changes in agricultural systems (FAO 2013:27). From the understandings of CSA above, it is clear that CSA is primarily concerned with adapting to a changing environment and reducing agricultural losses. This characteristic of CSA is aligned with the purpose of DRR.

CSA is therefore, effectively, adaptation in action and is now considered to be an accepted DRR strategy (Lei & Wang 2014:1590). Research has proven that the agricultural sector increasingly turns to climate-smart farming for more sustainable solutions to the impacts of climate change on the agricultural sector (FAO 2009:5–6; Lima 2014:29; Lipper et al. 2014:4). Climate change adaptation and mitigation and climate-smart crop production systems that ensure sustainability share common goals and work towards addressing climate hazards and reducing disaster risk. These processes aim to respond and adapt to changing climates by building resilience and practicing the sustainable utilisation of natural resources (Scherr, Shames & Friedman 2012:13). Thus, more effective and efficient DRR will positively assist the process of adaptation in the face of climate change (FAO 2013:417) and CSA does this by promoting sustainable agricultural systems (Lipper et al. 2014:3).

Because CSA can be considered an accepted and established tool for DRR within the context of climate change, it is important for all stakeholders to design region-appropriate CSA strategies. The most important element of successful adaptation to climate-smart farming, however, still lies in the hearts of the farmers who need to practice these changes (DEA 2015:69).

## Methodology

This research study applied a qualitative research approach, enabling the researchers to understand a complex phenomenon and to gain better insights from patterns emerging through collected data (Taylor & Bogdan 1998:7). As a starting point, the researchers conducted a comprehensive literature review to explore the research problem identified, hence the reason for this study to be exploratory and descriptive in nature. Secondly, an empirical investigation was conducted. A phenomenological (qualitative) approach was followed in an attempt to gain insight into the stakeholders’ experiences of the phenomenon of climate change because the most important reality is that which people perceive it to be (Taylor & Bogdan 1998:3, 11).

The sampling process was two-fold. The primary sampling process utilised for this study was stratified, purposeful sampling (Struwig & Stead 2001:123) targeting small-scale and commercial farmers and agricultural experts in the Mooifontein region. A secondary snowball sampling process was utilised (Struwig & Stead 2001:123) in order to support the first sampling process contributing to the depth of this study and adding more ‘information-rich cases’. The sampling size at the end of the data collection consisted of nine farmers (small-scale and commercial farmers) and six agricultural experts from the Mooifontein region. A saturation level was reached during the data collection process, and because of the richness of the data, this sample size can be regarded as representative of the Mooifontein farming community.

Maxwell (2013:102) states that research questions formulate what one aims to understand, but it is only through the data collection and interviewing process that one gains true understanding. ‘Qualitative researchers typically gather multiple forms of data’ (Creswell 2009:175) – as is also the case with this study. The data were collected through non-participant field observations, field notes, document studies, photographs and semi-structured personal interviews.

Non-participant observations, photographs and field notes were taken over a period of 3 years, equipping the researcher with rich insights regarding the impact of climate change on the Mooifontein farming community under study. A document study is particularly useful where historical events and experiences have relevance (De Vos et al. 2011:377). Historic rainfall records of the Mooifontein region were examined and personal diarised entries of the first official rainfall observer in this region were studied.

Semi-structured interviews were held with the research respondents as outlined earlier. Two separate sets of questionnaires were given for each target group (farmers and agricultural experts) to gain different perspectives and compare the views of the two target groups. The questionnaires consisted of open-ended questions, guiding the interview but allowing for participants to share their insights freely. The questionnaires also consisted of a risk perception index table, where participants expressed on a scale from ‘No risk at all’ to ‘Highest Risk’, their perspectives of perceived risks. These risks included a range of climatic, social and economic risks.

The qualitative data analysis was mainly based on a content analysis (Struwig and Stead 2001:14). All the collected data were transformed into meaningful content to produce the findings for this study – it is a process of digging deeper rather than ‘peeling back the layers of an onion’ (Creswell 2009:183). The findings of this study will be presented and discussed in the sections to follow.

## Results

The research aim of this study was to explore CSA as a resilience-building tool to ensure sustainable agricultural practices, specifically in the Mooifontein region. With the utilisation of the research methodology given, the findings of this study will be conveyed in two sections: Non-participant observations (findings from the field observations, field notes, document studies and photographs) and participants’ perspectives (findings from the semi-structured questionnaires).

### Non-participant observations

**FIGURE 1:**
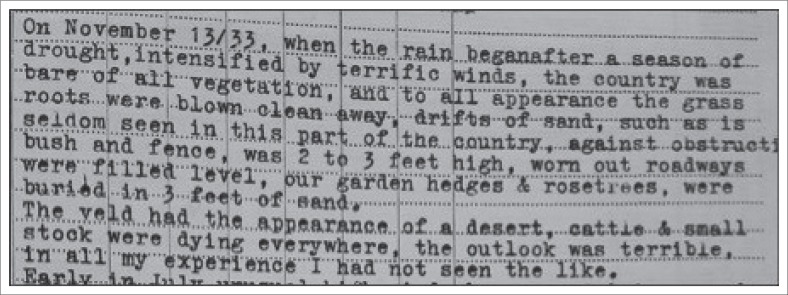
Dairy by Mr T.A. Young: The Miracle of 1933/1934.

**FIGURE 2:**
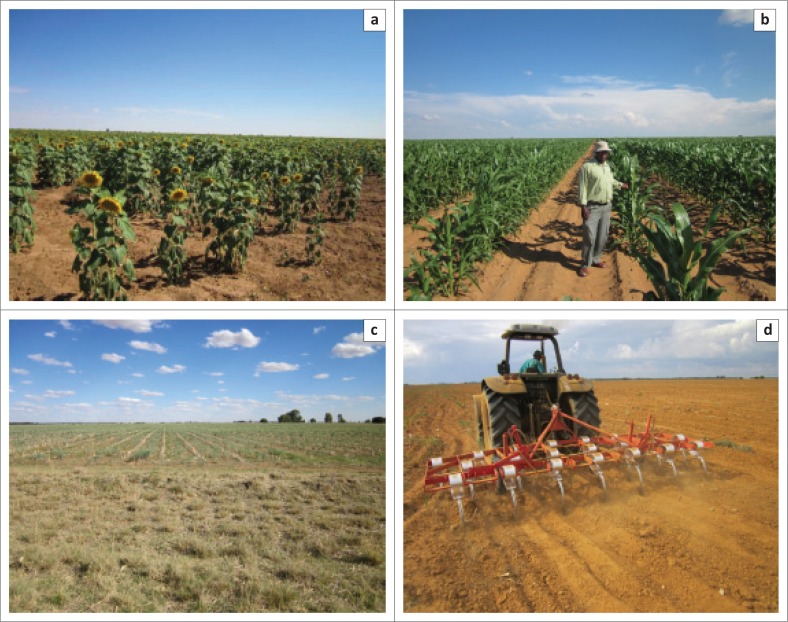
Photographs taken in 2013 in the Mooifontein farming region. (a) Sunflower field at Weltevrede, March 2013, (b) Maize growing in February 2013, Weltevrede, (c) Deelpan farmlands: Weed control is a widespread problem and (d) Vibroflexing is a step in the right direction.

**FIGURE 3:**
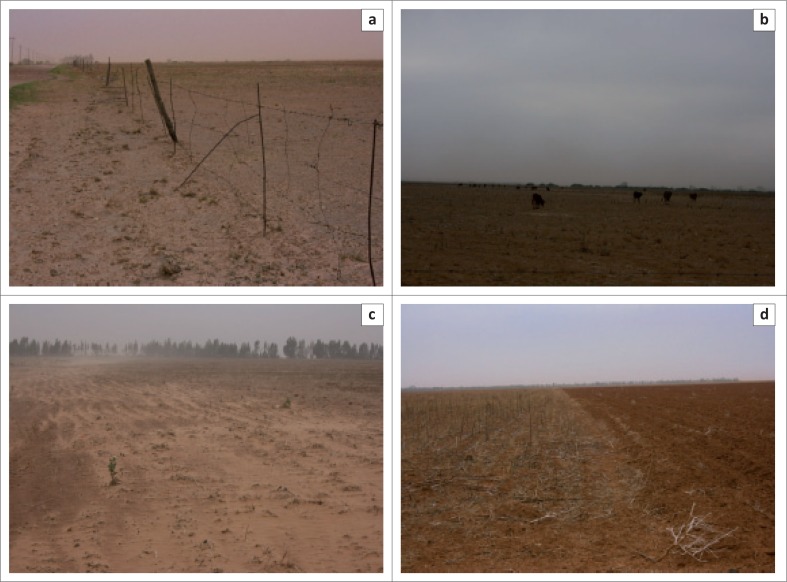
Photographs taken in 2016 in the Mooifontein farming region. (a) Dilapidated infrastructure near Brooksby is a common sight, (b) Thin herd animals scrounging for food in a drought-stricken cropping land near Enselsrust, (c) Ploughed lands wait for rain. The livestock have eaten all the dry matter left after the harvest, and (d) Cropping fields near Brooksby: the contrast between lands tilled by a mould-board plough (right) and no-tills lands (left).

The findings from the diary entry of the first South African Weather Services’ (SAWS) rainfall recorder, Mr T.A. Young in the ‘The Miracle of 1933/4’ is illustrated in Figure 1. An abstract of the diary can be viewed in Figure 1 which describes the climatic conditions as ‘terrific winds’, ‘drifts of sand’, hedges ‘buried in 3 feet of sand’, and ‘veld that had the appearance of a desert’. The livestock were dying and ‘the outlook was terrible’. The diarised entry can be compared to the situation in the Mooifontein farming region in 2015–2016. The first selection of photographs (Figure 2) was taken in 2013 by the researcher in the Mooifontein region. This creates a rich description of the farming region before the changing climatic conditions. The second set of photographs (Figure 3) was taken on a field trip on 08 October 2016. The effects of the 2015/2016 drought are evident in these pictures.

**FIGURE 4:**
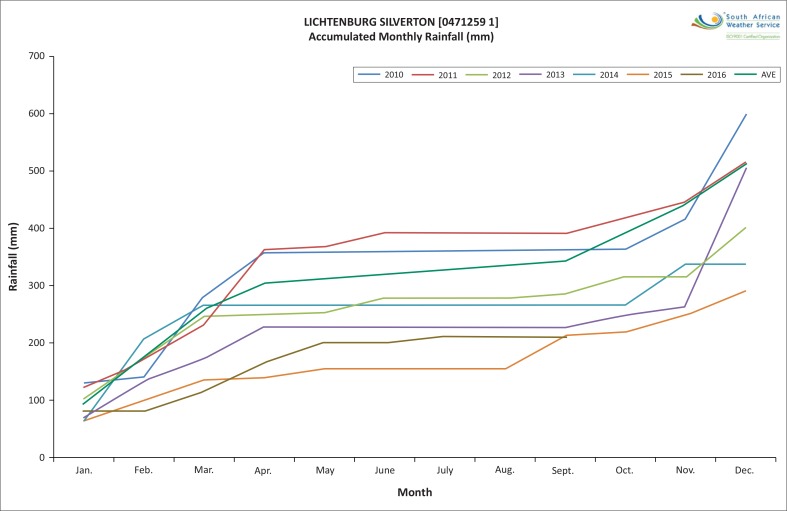
Accumulated monthly rainfall: Lichtenburg Silverton.

Additionally, a document study of the SAWS rainfall data was done for the Mooifontein region, which was recorded at the Lichtenburg Silverton (0471259 1) station (SAWS 2016). The findings reflected the total annual rainfall record from 1929 to 2016 of high and low rainfall, with an average rainfall of 515.5 mm. In spite of the cyclical nature of the results from SAWS, the results reflected a slowly decreasing trendline of the annual total rainfall, sliding from just below 600 mm/year in 1929 to 450 mm/year in 2016. The accumulated rainfall pattern from the year 2010–2016 shown in Figure 4 also reflects that the rainfall has been consistently below average. These results illustrate a true reflection of the current impact of the changing climatic conditions on this region.

### Perspectives from participants

The participants’ perceptions of climate change and other threats to sustainable farming in the Mooifontein region varied to a great extent. Some of the participants were sceptical: ‘I don’t buy into the theory of permanent climate change. I think our perceptions have changed, not the reality.’ Others believe that there have been changes, such as later onset of summer rainfall. Some of the farmer participants understood that climate change would force farmers to change ‘from tilling to minimum or no-till to retain soil moisture’, whilst others added that the last 3 years have been unpredictable: ‘We don’t know what to expect anymore.’ One adamant view from one of the participants was that nothing had changed and compared this to the dry cycle of the 1980’s, but ‘now the dry years seem to be one after the other’. Others did not understand what was meant with climate change.

The study found that the participating farmers preferred to think that the current experiences of climate changes were characteristic of a dry cycle that would pass. There was a prevailing scepticism about the theory of climate change, although it must be said this had not negatively influenced the farmers’ attitudes to adaptation as a tool for risk mitigation in this dry, hot region. All without exception were open to adopting new farming methods to mitigate climate risk. Most of the farmers agreed that the region was suited to maize production, with other cropping options being sunflowers, groundnuts, sugar beans, soybean and pastures. ‘We can adapt and continue growing cash crops. The pressure is from an economic rather than an environmental point of view.’ Challenges identified by the farmers included climate change, late rains, being forced to plant later with the risk of frost on immature crops, high temperatures that dry the soil and scorch plants, theft of crops, animals grazing in the crops and low market prices.

**FIGURE 5:**
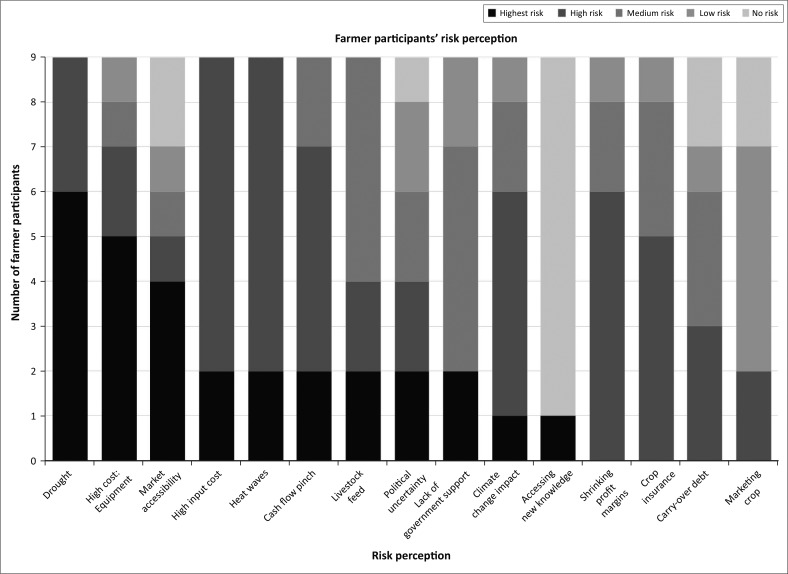
Risk Perception Index of farmer participants.

**FIGURE 6:**
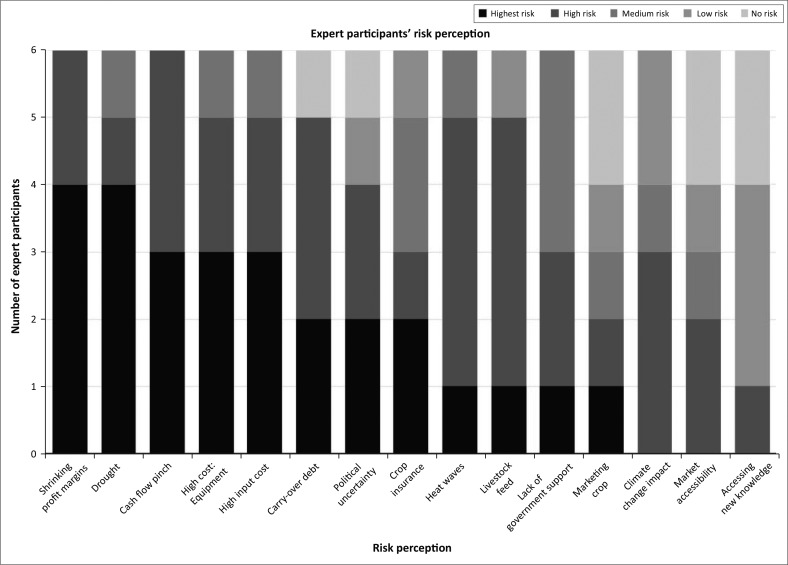
Risk Perception Index of expert participants.

The results from the farmer participants and the agricultural experts regarding the Risk Perception Index (RPI) will be presented in separate figures (see Figures 5 and 6).

The findings from the farmers’ RPI reflect the different stressors that farmers experience depending on each one’s unique circumstances. Figure 5 reflects that the risks perceived by the farmers as the highest risks include drought, accompanied by high temperatures, economic pressures from the high cost of inputs, costs of machinery and shrinking profit margins. A noteworthy result from this index is the fact that the ‘lack of government support’ is perceived to be a medium risk. This finding can be prescribed to the independence and the resilience of farmers, for they are still able to cope under prevailing circumstances without external support.

The RPI of the agricultural experts reveals different findings to those of the farmers. The findings revealed that the experts perceive political uncertainty as a higher risk in relation with the farmers’ perceptions. The findings in Figure 6 confirms that economic stressors like the shrinking profit margins, cash flow pinch and high costs are a perceived concern rated as a high risk. In addition, climate stressors like drought and heat waves are also rated amongst the highest risks on the index.

The findings on the farmers’ insights into CSA and sustainable farming practices reflected that the large-scale farmers were all aware of climate-smart farming systems and many felt they had already begun implementing CSA by shifting to climate adaptation and greater use of information systems and tools offered by modern technology:

For the past 30 years I have been working IN nature, but over the last 5-8 years I have changed my approach and now I am looking for opportunities to work WITH nature by exploiting natural resources, but keeping everything in a sustainable balance.

Most of the small-scale farmers were unsure what the term ‘climate-smart’ meant and some surmised it had to do with changing tillage systems and conserving soil moisture.

All the farmers agreed that soil moisture conservation and health of crops were critical. However, there were mixed views on whether no-till would succeed in the long term, with strong opinions held on both sides of the fence. Some said they were not convinced by what they had seen in the region so far: ‘The biggest no-till farmer in our area is dealing with huge challenges’ – and thus have opted for a minimum till approach together with other precision farming practices. The problem is that none of the farmers can afford to risk experimenting because of their small profit margins and high cost of new tractors and implements. The only fully committed no-till farmer stated that no-till farming required excellent weed control, which is expensive. This participating farmer acknowledged that costs had been incurred in the process of adaptation; however, the farmer insisted that ‘the cost savings have been significant’. Precision farming is considered key to future efficiency because the information gathered and the precision with which seeds, chemicals and fertilisers could be applied cuts costs significantly. Other adaptations described by the participants were seed population, accurate fertilisation using soil samples, the use of Roundup Ready^®^ seed and Roundup chemicals for weed control, ripping soils deeper so that moisture can filter deeper, plough less, use newest spray technologies and the elimination of weeds.

With the results of this study discussed in the above sections, the next section will further discuss the findings of this study.

## Discussion

The findings of this study confirm that the Mooifontein region is prone to dry spells and extremely high temperatures. This is significant because most of the agricultural land is based on rain-fed agricultural systems. This study therefore agrees with Vogel (2008) that farmers in this region should adapt to CSA practices to secure more sustainable production and building on farmers’ resilience. The findings support that there is a wide range of stressors that threaten sustainable agriculture in the Mooifontein region. The participants in this study had differing opinions of climate change. Some had a vague understanding of the concept of climate change – others again perceived it as generational belief such as cyclical patterns of climate. Despite the different opinions, all participants agreed that the climate has changed, and the findings bode well with the literature that proves that the climate is indeed changing and placing agricultural activities at risk (DEA 2013:120–121, 2015:67; O’Brien et al. 2008:10; Speranza 2010:16; Wisner et al. 2012:207). This study further revealed that the pressures found in the Mooifontein region are not only prescribed to climate change. Speranza (2010:16) and Reid and Vogel (2006:196) stated that many farmers were already trapped in difficult predicaments, making them vulnerable and enhancing their struggle to cope with risks and stressors. Poor infrastructure, crop theft, overgrazed land, death of livestock and marauding animals were listed as some of the stressors experienced by the farmers in the area of study. Many of the changes ascribed to the changing climate, for example, poor yields and seasonal unpredictability were highlighted by participants, already threatening sustainable agriculture in the Mooifontein region.

Mitchell et al. (2010:2) and FAO (2013:360) state the critical links between climate change and DRR in the context of farmer development and livelihood vulnerability. Pettengell (2010:33) further states that an ideal development programme would simultaneously address climate risk and DRR because failure in doing so would jeopardise development initiatives. The findings of this study revealed a widespread consensus amongst stakeholders that climate is indeed changing, placing agricultural activities at risk (Wisner et al. 2012:207). The regional information for the NWP in which the field of study lies (DEA 2013:120–121) stated that long-term predictions for the region of study was a steady drying from 2015 to 2035 and that annual temperatures would increase by up to 2.5 °C. The literature review highlights that the African continent was likely to feel the impacts of climate change more severely than others (O’Brien et al. 2008:10), in particular where communities were already vulnerable as a result of poverty, sickness and marginalised livelihoods (Speranza 2010:16). It was found that change was already experienced and the summer rains were starting later and the ‘planting window’ was later (DEA 2015:67).

A CSA approach can therefore offer solutions to the agricultural sector of the Mooifontein region. This study supports this view that the farmland in this region is comprised of inherently high-quality rangelands and deep soils, which are well suited for cropping. Despite many farmers’ lack of knowledge with regard to CSA approaches, they did demonstrate a willingness to embrace adaptation and agreed that sustainable farming systems were identified as a need to be further investigated in order to improve the agricultural sector’s resilience to ensure more sustainable agricultural sectors in the region.

## Conclusion and the way forward

This study explored the impacts of climate change on the agricultural sector of the Mooifontein region and suggests that CSA is a tool for moving towards sustainability. The farmers in the Mooifontein region are experiencing many pressures. This is a region that is vulnerable to climate risk; however, the farmers in this region proved to be a resilient community. Although much can still be learned about CSA and CCA, it is suggested that there are existing strategies that could be employed towards building resilience and ensuring that the best yields possible are produced under prevailing circumstances. Appropriate CSA practices need to be identified and practiced for this community of farmers to build on their resilience and to ensure a more sustainable agricultural sector. To do so, national policy needs to be more effectively institutionalised at the provincial and municipal levels and budgets need to be allocated towards sustainable development initiatives. In doing so, the agricultural sector in the Mooifontein region could once again become a rewarding sector contributing towards the sustainability of this region. Broader investigation into the stressors threatening the viability of crop farming in communal farming areas such as the Mooifontein region needs to be conducted. However, there is further scope for research on CSA in this region, focusing on barriers to effective extension services ideally positioned at knowledge transfer. This study recommends that an effective education programme for knowledge transfer of CSA to farmers should be further explored.
